# A systematic review and meta-analysis of the association between vitamin D and ovarian reserve

**DOI:** 10.1038/s41598-021-95481-x

**Published:** 2021-08-06

**Authors:** Elham Karimi, Arman Arab, Masoumeh Rafiee, Reza Amani

**Affiliations:** 1 Department of Clinical Nutrition, School of Nutrition and Food Science, Food Security Research Center, Isfahan University of Medical Sciences, Isfahan, Iran; 2 Research Development Center, Arash Women’s Hospital, Tehran University of Medical Sciences, Tehran, Iran; 3 Department of Community Nutrition, School of Nutrition and Food Science, Food Security Research Center, Isfahan University of Medical Sciences, Isfahan, Iran

**Keywords:** Endocrinology, Medical research

## Abstract

It is hypothesized that vitamin D deficiency could be related to ovarian reserve. This systematic review and meta-analysis was undertaken to analyze the possible association between vitamin D and ovarian reserve among adolescent and adult women. All eligible studies identified through the ISI Web of Science, PubMed, and Scopus were included up to May 2021. A random-effects meta-analysis model was implemented and a weighted mean difference (WMD) and 95% confidence interval (CI) were calculated. A total of 38 papers covering 8608 individuals were enrolled in this systematic review and meta-analysis. Antral follicle count (AFC) was significantly lower among Asians (WMD − 0.65; 95% CI − 1.28 to − 0.01; P = 0.04; I^2^ = 0.0%) and luteinizing hormone (LH) levels were higher in non-Asians (WMD 2.16 IU/L; 95% CI 0.20 to 4.12; P = 0.031; I^2^ = 9.3%) with vitamin D insufficiency/deficiency. Also, there was a negative correlation between vitamin D and LH/FSH ratio in women with normal body mass index (BMI) (Fisher’s Z: − 0.18; 95% CI − 0.37 to − 0.008; P = 0.041; I^2^ = 51.5%). Although there were no significant associations between serum vitamin D levels and any of the intended ovarian reserve markers, subgroup analyses have found significant findings regarding AFC, LH, and LH/FSH ratio. In order to understand the underlying mechanisms of vitamin D in female reproduction, further attempts are needed.

## Introduction

Vitamin D is an essential nutrient with a hormone-like activity that was initially recognized for its importance in bone health and calcium–phosphate homeostasis^1^. Though, the recent vitamin D deficiency pandemic has emphasized other functions^2^. Growing documents suggest that vitamin D deficiency upsurges the risk of various chronic disorders including obesity, type 1 diabetes mellitus, cardiovascular, infectious, and autoimmune diseases; certain types of cancer; depression, and chronic pain^2^.

More recently, a regulatory role for vitamin D has been suggested in female fertility^3,4^. In this context, previous epidemiological investigations have proposed a seasonality in female reproductive capacity which might be explained partially by seasonal variation in serum levels of vitamin D^5^. Biological activities of vitamin D are applied through the vitamin D receptors (VDR) that have been detected in the ovary especially in granulosa cells and theca cells, endometrium and placenta^6^. This diverse VDR expression proposes a potential role of vitamin D in female reproduction^7^. Though the underlying mechanism by which vitamin D may involve in reproductive physiology is poorly known, a direct link between vitamin D and ovarian steroidogenesis has been proposed. This link is derived from several in-vitro and in-vivo studies indicating that vitamin D could stimulate steroidogenesis in ovarian cells by modulating the mRNA and protein expression levels of steroidogenic enzymes including Cyp11a1, StAR, Cyp19a1, and 3β-HSD^8,9^. Reproductive potential of an individual is mainly explained by the quality and the quantity of ovarian primordial follicles that was diminished as women get older. Therefore, several markers have suggested to illustrate the ovarian reserve status. Low anti-Mullerian hormone (AMH), low antral follicle count (AFC), low luteinizing hormone (LH), high follicle-stimulating hormone (FSH), and low LH/FSH ratio may represent a diminished ovarian reserve status^10^.

Nevertheless, the findings of experimental studies are consistent enough to suggest the association between vitamin D and ovarian reserve, the evidence of human studies are commonly inconsistent, with some documents supporting this relation^11,12^ and others failing to detect any significant association^7,13,14^. For example, Dennis et al.^11^ have suggested that vitamin D may pose a regulatory role in the production of AMH; however, the works of Drakopoulos et al.^7^, Pearce et al.^13^, and Shapiro et al.^15^ did not verify this association. With regard to the conflicting findings and the increasing trend of interest about the role of vitamin D in female reproduction, this study collects the available documents to clarify this issue. We aimed to perform a systematic review and meta-analysis to reach a firm conclusion about the possible link between serum vitamin D levels and ovarian reserve markers including AMH, AFC, LH, and FSH among adolescent and adult women using observational studies.

## Methods

The present study was designed and conducted based on the Preferred Reporting Items for Systematic Reviews and Meta-Analysis (PRISMA) Statements^16^ and also was registered (Prospero database: CRD42020191703).

### Data source and search strategy

The electronic databases ISI Web of Science, PubMed, and Scopus were systematically searched from the earliest available date to May 2021 to identify relevant studies. Two investigators (A.A and E.K) independently searched the above-mentioned databases to find studies on the association between vitamin D and ovarian reserve, using the following keywords: (“ovarian reserve” OR “oocyte reserve” OR “Anti Mullerian hormone” OR “Mullerian inhibiting factor” OR “anti Mullerian factor” OR “Mullerian inhibitory substance” OR “Mullerian inhibiting hormone” OR “Mullerian inhibiting substance” OR “Mullerian regression factor” OR “AMH” OR “Follicle-stimulating hormone” OR “FSH” OR “Luteinizing hormone” OR “LH” OR “Antral follicle count” OR “AFC”) AND (“vitamin D” OR “25-Hydroxyvitamin D” OR “cholecalciferol” OR “ergocalciferol” OR “calciol” OR “vitamin D3” OR “25(OH)D3”).

The bibliographic lists of any of the eligible studies were also scanned to detect any additional qualified ones. We also contacted expert scientists in the field of ovarian reserve and vitamin D to lower the chance of missing any additional studies.

### Study selection and eligibility criteria

The PICO (Population/intervention/comparison/outcome) components were as follows: **P** (adolescent and adult premenopausal women with vitamin D deficiency/insufficiency), **I** (serum levels of vitamin D), **C** (women with a normal level of serum vitamin D), **O** (ovarian reserve markers including AMH, AFC, LH, FSH, and LH/FSH ratio). The inclusion criteria were as follows: (1) original human observational studies either with case–control, cross-sectional, or longitudinal design; (2) published in the English language; (3) assessed serum levels of at least one of the ovarian reserve markers including AMH, AFC, LH, FSH, and LH/FSH ratio in association with 25(OH)D; and (4) those presented as (4.1) comparison of ovarian reserve markers (AMH, AFC, LH, FSH, and LH/FSH ratio) between women with vitamin D insufficiency/deficiency and vitamin D sufficient ones; or (4.2) correlation between 25(OH)D and ovarian reserve markers (AMH, AFC, LH, FSH, and LH/FSH ratio).

The exclusion criteria were as follows: (1) Experimental studies; (2) recruited pregnant, lactating, or postmenopausal women; and (3) poster abstracts, case reports, review articles, editorials, and non-original full-length articles, or those without original data or articles with no appropriate outcome measures. Two assessors independently (A.A and E.K) conducted the selection process. Any disagreement was resolved through discussion with a third reviewer (R.A).

### Data extraction

The following data were extracted: first author's name, year of publication, geographical location, sample size, participant characteristics including health status, age and body mass index (BMI), 25(OH)D assay method, cut-off values of vitamin D status, the season of sample collection, study design, reported ovarian reserve markers, and statistical adjustment.

### Quality assessment

The quality assessment of eligible studies was performed by two reviewers (A.A and E.K) individually using the Newcastle–Ottawa Scale (NOS) star system (ranged, 0–9 stars)^17^, which focuses on selection, comparability, and outcome. Studies scoring ≥ 7, 4–6, and ≤ 3 points were assumed as high, moderate, and low quality, respectively^18^.

### Statistical analysis

The present study was performed to present the association between vitamin D and ovarian reserve quantitatively. Prior to the calculation of the effect size, the concentration of AMH was converted to ng/mL, LH to IU/l, and FSH to IU/l. In the current study, we calculated two types of effect sizes: (1) weighted mean difference (WMD) in AMH, AFC, LH, or FSH between vitamin D insufficiency/deficiency and sufficient vitamin D groups; and (2) Fisher’s Z of the correlation between 25(OH)D and AMH, AFC, LH, FSH or LH/FSH. If a document provided the results stratified by certain variables like age, BMI, and participants’ health status, it was divided into two different studies supposed to be independent of each other. In the absence of the mean and standard deviation (SD), values of median and range or median and interquartile range were converted into mean and SD based on related formulas^19^. Fisher’s Z and its SE using correlation coefficients (r) and sample size (N) were calculated by the relevant formula^20^. Heterogeneity between effect size of included studies was estimated by chi-squared (χ^2^) test and I^2^ statistic [*I*^2^ index < 40 (low heterogeneity), 40–75 (moderate heterogeneity) and > 75% (high heterogeneity)]^21^. When there was no significant heterogeneity, the effect size was calculated using a fixed-effects model. Otherwise, a random-effects model was used^22^. Subgroup analyses were done based on different characteristics of included studies, whenever possible, to check the sources of heterogeneity. Publication bias was assessed using Egger’s and Begg’s statistics^23^ and in the presence of significant publication bias, trim & fill analysis was performed to detect any possibly missed study. The sensitivity analyses were also conducted to evaluate the influence of every single study on the stability of the meta-analysis findings. The statistical analyses were done using STATA statistical program version 11.2 (Stata Corporation, College Station, TX, USA). A two-sided p-value < 0.05 was considered statistically significant.

### Ethical approval

All analyses were based on previous published studies; thus, no ethical approval was required.

## Results

### Characteristics of included studies

**Figure 1:**
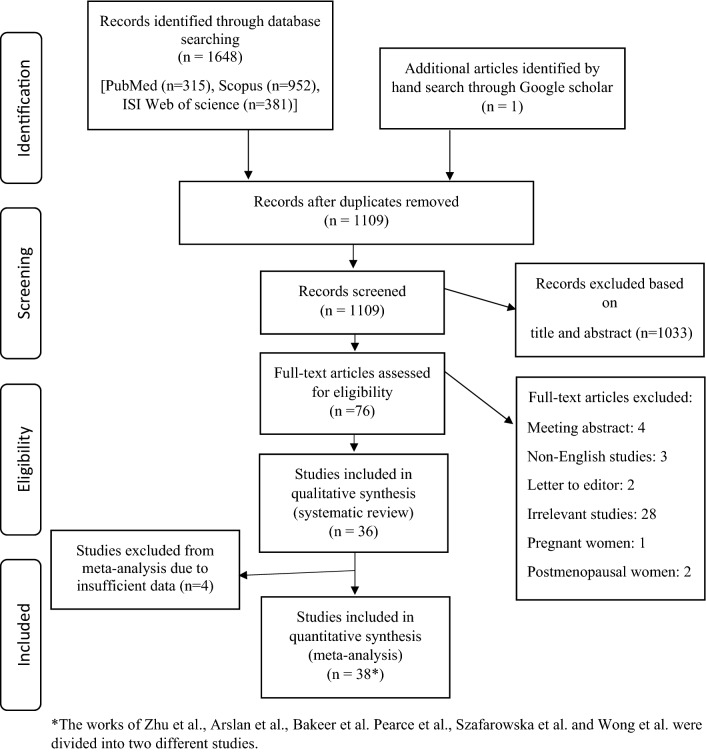
The flow diagram of study selection.

The primary search yielded 1648 articles. A total of 36 eligible articles involving 7882 individuals were included in this study with a sample size ranging from 26 to 851. Participants' mean age ranged from 17.8 to 42.5 years old and BMI from 20.7 to 35.7 kg/m^2^. The enrolled studies were published between 2009 and 2020 of which 9 were from Turkey^24–32^, 5 from United States^12,15,33–35^, 3 from Iran^36–38^, 3 from Poland^39–41^, 2 from South-Korea^14,42^, 2 from Saudi Arabia^43,44^, 2 from China^45,46^, 2 from India^47,48^. Others were from Spain^49^, Belgium^7^, Slovakia^50^, Egypt^51^, Bosnia and Herzegovina^52^, Australia^53^, Japan^54^, and Canada^55^. Moreover, 21 studies were cross-sectional in design^7,14,24–29,33,36,37,39,40,42,43,46,47,49,51,52,54^, 9 case–control^30–32,38,41,44,45,48,50^ and 6 cohorts^12,15,34,35,53,55^. Seventeen studies mentioned the season of sample collection^7,14,15,27,32–35,37,38,40–42,52–55^. Twenty-five studies selected serum vitamin D < 20 ng/ml as deficient, eight studies serum vitamin D < 10 ng/ml, and the others did not mention the cut-off values. Based on the NOS, 23 studies were ranked as high quality^7,12,15,24,27–29,32–35,37,42–46,48–50,53–55^ and 13 moderate^14,25,26,30,31,36,38–41,47,51,52^, respectively. Table 1 provides the primary information of enrolled studies and Fig. 1 provides the study selection process applied for this systematic review and meta-analysis.

**Table 1:** Characteristics of included studies.

Author, Year	Location	Sample size	Age (Mean)	BMI (kg/m^2^)	Study Design	Women with ovarian dysfunction	Vit D assay method	Season of sample collection	Adjustments	Cut-off values of vitamin D status (ng/mL)	Outcome	Quality assessment score
Yildizhan et al., 2009	Turkey	100	26.09	27.50	Cross-sectional	Yes	HPLC	NM	–	Deficient (< 20), insufficient (20–30), sufficient (> 30)	LH/FSH	High
Kulaksizoglu et al., 2013	Turkey	76	36.95	29.95	Cross-sectional	Yes/No	HPLC	NM	–	Deficient (< 10), insufficient (10–20), sufficient (> 20)	FSH	Moderate
Kebapcilar et al., 2013	Turkey	63	37.2	29.4	Cross-sectional	Yes/No	HPLC	NM	–	Deficient (< 10), insufficient (10–20), sufficient (> 20)	FSH, LH	Moderate
Kozakowski et al., 2014	Poland	26	28.4	35.7	Cross-sectional	Yes	CLIA	NM	–	Deficient (< 20), insufficient (20–30), sufficient (> 30)	LH/FSH	Moderate
Chang et al., 2014	South Korea	73	33.8	20.7	Cross-sectional	No	RIA	Winter	–	Deficient (< 10), insufficient (10–20), sufficient (> 20)	AMH, AFC, FSH	Moderate
Velija-Asimi et al., 2014	Bosnia and Herzegovina	60	26	25.88	Cross-sectional	Yes	RIA	Autumn, Winter	–	Deficient (< 20), insufficient (20–30), sufficient (> 30)	FSH, LH, LH/FSH	Moderate
Jukic et al., 2015	US	527	42	NM	Cross-sectional	Yes	RIA	All	Age, Education, Race, BMI, Alcohol Intake, Smoking, Physical activity, Age at menark, Gravidity, Mother’s age at menopause, Season	Deficient (< 20), insufficient (20–30), sufficient (> 30)	FSH	High
Ersoy et al., 2016	Turkey	130	32.85	25.2	Cross-sectional	Yes/No	ELISA	Winter	–	Deficient (< 20), insufficient (20–30), sufficient (> 30)	FSH	High
Drakopoulos et al., 2016	Belgium	283	32.2	23.5	Cross-sectional	Yes	ELISA	All	Age, BMI, smoking status, infertility cause and season of blood sampling	Deficient (< 20), insufficient (20–30), sufficient (> 30)	AMH, AFC	High
Fabris et al., 2017	Spain	851	25	22.63	Cross-sectional	No	CL	NM	–	Deficient (< 20), insufficient (20–30), sufficient (> 30)	AMH, AFC	High
Zhu et al., 2017	China	109	30.2	21.05	Cross-sectional	No	CS	NM	–	–	AMH	High
Kim et al., 2017	South Korea	291	42.5	20.8	Cross-sectional	No	RIA	Spring, Winter	Age	Deficient (< 10), insufficient (10–20), sufficient (> 20)	AMH	High
Arefi et al., 2018	Iran	189	32.21	26.7	Cross-sectional	Yes	ELISA	NM	–	Deficient (< 20), insufficient (20–30), sufficient (> 30)	AFC	Moderate
Daghestani et al., 2018	Saudi Arabia	88	24.74	22.48	Cross-sectional	Yes/No	ELISA	NM	–	–	FSH, LH/FSH	High
Bakeer et al., 2018	Egypt	70	26.1	27.47	Cross-sectional	Yes/No	ELISA	NM	–	–	AMH	Moderate
Wong et al., 2018	Japan	695	30.33	22.25	Cross-sectional	Yes/No	CLIA	All	BMI	Deficient (< 20), insufficient (20–30), sufficient (> 30)	AMH, AFC	High
Arslan et al., 2019	Turkey	146	28.6	26.1	Cross-sectional	Yes	NM	NM	–	Deficient (< 20), insufficient (20–30), sufficient (> 30)	AMH, LH, FSH	High
Bednarska-Czerwinska et al., 2019	Poland	53	34.7	22.2	Cross-sectional	Yes	ECLIA	All	–	Deficient (< 20), insufficient (20–30), sufficient (> 30)	AMH	Moderate
Inal et al., 2020	Turkey	240	29.09	25.46	Cross-sectional	Yes	LC–MS	NM	–	Deficient (< 20), insufficient (20–30), sufficient (> 30)	FSH, LH, AFC	High
Alavi et al., 2020	Iran	287	29.95	25.11	Cross-sectional	Yes	ELISA	Summer, Autumn	Age, BMI	Deficient (< 20), insufficient (20–30), sufficient (> 30)	AMH	High
Lata et al., 2017	India	70	18–40	NM	Cross-sectional	Yes/No	CLIA	NM	–	Deficient (< 10), insufficient (10–20), sufficient (> 20)	AMH	Moderate
Ghadimi et al., 2014	Iran	192	17.85	NM	Case–control	Yes/No	CLIA	Winter	–	Deficient (< 10), insufficient (10–30), sufficient (> 30)	LH, FSH	Moderate
Figurova et al., 2016	Slovakia	165	28.94	24.75	Case–control	Yes/No	RIA	NM	Age	Deficient (< 10), insufficient (10–30), sufficient (> 30)	LH, LH/FSH	High
Yilmaz et al., 2015	Turkey	140	22.86	NM	Case–control	Yes/No	LC–MS	NM	–	Deficient (< 20), insufficient (20–30), sufficient (> 30)	LH, FSH	Moderate
Ganie et al., 2016	India	168	23.80	22.31	Case–control	Yes/No	RIA	NM	Age, BMI	Deficient (< 20), insufficient (20–30), sufficient (> 30)	LH, FSH	High
Bostanci et al., 2018	Turkey	66	18.44	22.58	Case–control	Yes/No	ELISA	NM	–	Deficient (< 20), insufficient (20–30), sufficient (> 30)	LH/FSH	Moderate
Kensara et al., 2018	Saudi Arabia	128	31	21.8	Case–control	Yes/No	ELISA	NM	Age, BMI	Deficient (< 20), insufficient (20–30), sufficient (> 30)	FSH, LH	High
Kokanali et al., 2019	Turkey	385	25.24	27.47	Case–control	Yes	ELISA	Spring	–	Deficient (< 20), insufficient (20–30), sufficient (> 30)	FSH, LH, LH/FSH, AMH	High
Szafarowska et al., 2019	Poland	98	33.9	21.65	Case–control	Yes/No	ELISA	All	–	Deficient (< 20), insufficient (20–30), sufficient (> 30)	AMH	Moderate
Xu et al., 2019	China	105	18–40	NM	Case–control	Yes/No	MS	NM	Age, BMI, education, annual household income	Deficient (< 20), insufficient (20–30), sufficient (> 30)	FSH, AMH	High
Merhi et al., 2012	US	388	37.44	29.23	Cohort	No	LC–MS	NM	HIV status, BMI, race/ethnicity, smoking history, current illicit drug use, fasting glucose and insulin levels, EGFR, and geographic site of participation	Deficient (< 20), insufficient (20–30), sufficient (> 30)	AMH	High
Garbedian et al., 2013	Canada	173	34.5	24.05	Cohort	Yes	NM	Spring, Summer, Autumn	–	Deficient (< 20), insufficient (20–30), sufficient (> 30)	AFC, FSH	High
Pearce et al., 2015	Australia	340	32.1	26.1	Cohort	Yes/No	CLIA	All	Age, BMI	Deficient (< 10), insufficient (10–20), sufficient (> 20)	AMH, AFC	High
Jukic et al., 2018	US	561	34.58	33.52	Cohort	No	LC–MS	All	Age, Race, smoking History, BMI , Recent use of hormonal birth control	Deficient (< 20), insufficient (20–30), sufficient (> 30)	AMH, FSH	High
Shapiro et al., 2018	USA	457	39.45	24.9	Cohort	Yes	CLIA	All	Age, BMI, seasonal variation	Deficient (< 20), insufficient (20–30), sufficient (> 30)	FSH, AMH	High
Harmon et al., 2020	USA	89	28	24	Cohort	No	ELISA	All	Age, BMI, Physical Activity, Parity status	Deficient (< 20), insufficient (20–30), sufficient (> 30)	FSH, LH	High

*HPLC* high-performance liquid chromatography, *CLIA* chemiluminescent immunoassay, *RIA* radio immunoassay, *ELISA* enzyme-linked immunosorbent assay, *CL* chemiluminescence, *CLMI* chemiluminescent microparticle immunoassay, *ECLIA* electrochemiluminescence immunoassay, *CMIA* chemiluminescent microparticle immuno assay, *LC–MS* liquid chromatography–mass spectrometry, *MS* mass spectrometry, *CS* chemical spectrophotometric, *PCOS* polycystic ovary syndrome, *POI* primary ovarian insufficiency, *POF* premature ovarian failure, *NM* not mentioned, *BMI* body mass index, *EGFR* estimated glomerular filtration rate, *AMH* anti mullerian hormone, *AFC* antral follicle count, *LH* luteinizing hormone, *FSH* follicle stimulating hormone.

### Findings from the systematic review

Four studies have examined the association between 25(OH)D and ovarian reserve markers, however, due to insufficient data, they were described qualitatively.

In the first study, Ghadimi et al.^38^, have assessed the association between vitamin D and metabolic parameters of PCOS in high-school girls (mean age: 17.85 years old) through a case–control design. The study included 104 PCOS individuals and 88 non-PCOS controls. Based on Pearson’s test, no significant correlation was found between 25(OH)D levels and LH and FSH levels.

The other investigation has been conducted by Jukic et al.^33^ in 2015 to explore the relationship between FSH and serum vitamin D among 527 premenopausal women (mean age: 17.85 years old). In this cross-sectional study, 25(OH)D and urinary FSH levels were inversely correlated (P = 0.003).

The other study in 2018 was conducted by Arefi et al.^36^ to explore the correlation between vitamin D deficiency and ovarian reserve through a cross-sectional study of 189 Iranian infertile women (mean age: 32.21, mean BMI: 26.7 kg/m^2^). The result of this study proposed a highly significant correlation between vitamin D and AFC (p < 0.001).

The last evidence was conducted among infertile and fertile females (18–40 years old) to investigate the correlation of vitamin D deficiency with serum AMH. The result of this cross-sectional study failed to show any significant correlation between vitamin D and AMH in either fertile or infertile women^47^.

### Findings from meta-analysis

#### Comparison of ovarian reserve markers between women with vitamin D insufficiency/deficiency and sufficient ones

##### Serum 25(OH)D levels and AFC

**Figure 2:**
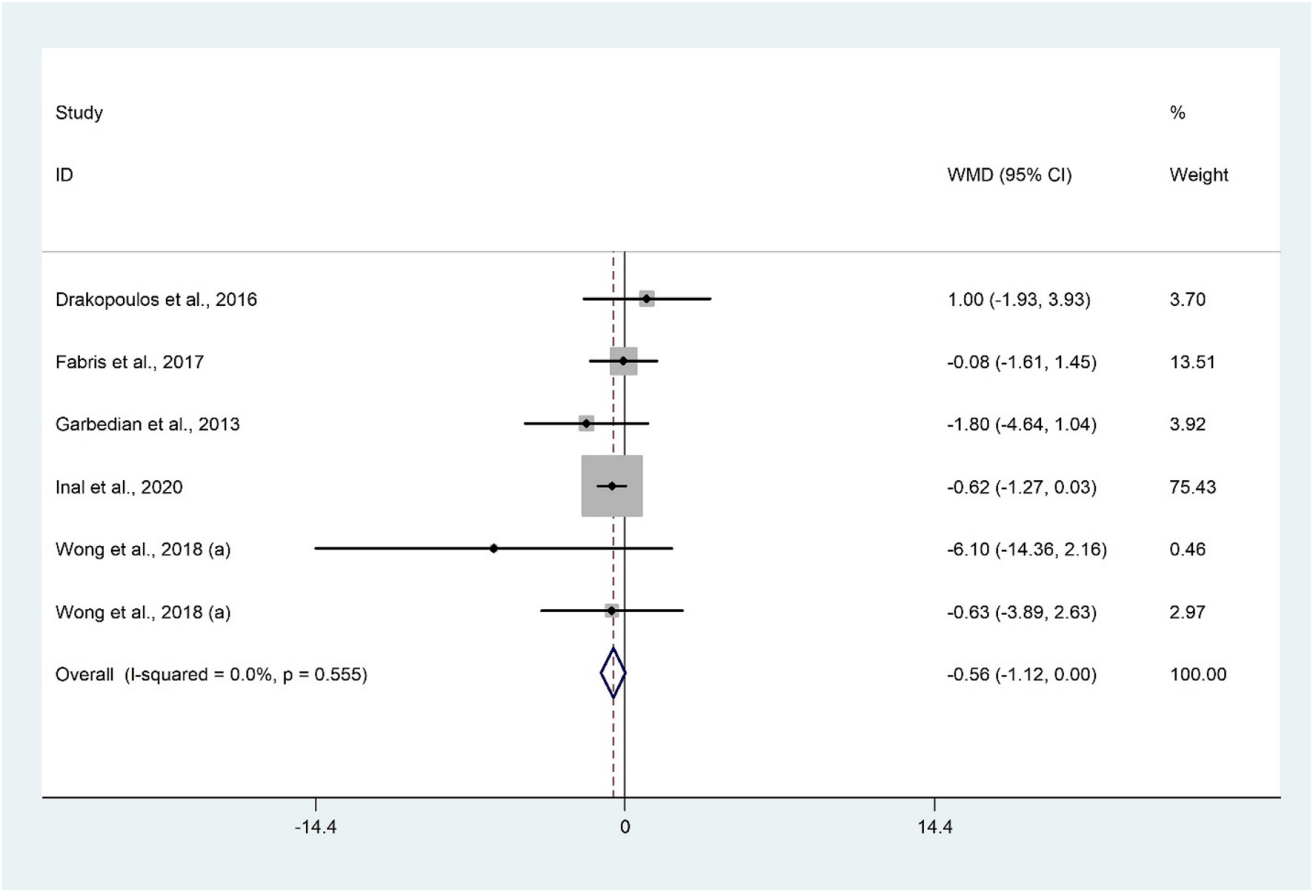
Forrest plot of the comparison of the AFC between women with vitamin D insufficiency/deficiency and sufficient ones.

The analysis of six studies^7,29,49,54,55^ with 2242 participants revealed that AFC is lower in patients with vitamin D insufficiency/deficiency compared to their controls (WMD − 0.56; 95% CI − 1.12 to − 0.004; P = 0.052) without significant heterogeneity (I^2^ = 0.0%, P = 0.555) (Fig. 2). Subgroup analysis revealed a significant result only among Asian population (WMD − 0.65; 95% CI − 1.28 to − 0.01; P = 0.04) (Table 2). Findings from sensitivity analysis revealed that the exclusion of Drakopoulos et al.^7^ (WMD = − 0.61; 95% CI − 1.19 to − 0.04) and Fabris et al.^49^ (WMD = − 0.63; 95% CI − 1.23 to − 0.02) studies from the analysis changed the overall result.

**Table 2:** Subgroup analysis of the differences in ovarian reserve markers between women with vitamin D insufficiency/deficiency and controls.

Sub-grouped by	No. of studies	Effect size^1^	95% CI	I^2^ (%)	P for heterogeneity	P for between subgroup heterogeneity
**AFC**						
Ovarian dysfunction						**0.557**
Without ovarian dysfunction	2	− 0.17	− 1.56, 1.20	0.0	0.765	
With ovarian dysfunction	4	− 0.66	− 1.73, 0.40	15.0	0.317	
Geographical population						**0.529**
Asian	3	− 0.65	− 1.28, − 0.01	0.0	0.431	
Non-Asian	3	− 0.21	− 1.43, 1.01	0.0	0.389	
**AMH**						
Ovarian dysfunction						**0.386**
Without ovarian dysfunction	2	− 0.44	− 1.46, 0.58	52.0	0.149	
With ovarian dysfunction	6	0.13	− 0.48, 0.75	69.1	0.006	
Geographical population						**0.127**
Asian	4	− 0.97	− 2.45, 0.50	63.5	0.042	
Non-Asian	4	0.26	− 0.34, 0.87	64.6	0.037	
**FSH**						
Geographical population						**0.129**
Asian	4	0.04	− 0.58, 0.67	72.0	0.013	
Non-Asian	3	− 0.28	− 0.80, 0.23	0.0	0.745	
Participants BMI						**0.518**
Overweight/obese	4	− 0.09	− 0.78, 0.60	72.5	0.012	
Normal	3	− 0.05	− 0.56, 0.46	12.7	0.318	
**LH**						
Geographical population						**0.015**
Asian	4	− 0.21	− 0.59, 0.16	0.0	0.740	
Non-Asian	2	2.16	0.20, 4.12	9.3	0.294	
Participants BMI						**0.527**
Overweight/obese	4	0.03	− 0.87, 0.94	57.9	0.068	
Normal	2	0.36	− 1.18, 1.91	0.0	0.383	

*AFC* antral follicle count, *AMH* anti-Mullerian hormone, *FSH* follicle stimulating hormone, *LH* luteinizing hormone, *BMI* body mass index.

^1^Calculated by Random-effects model as weighted mean difference.

##### Serum 25(OH)D levels and AMH

**Figure 3:**
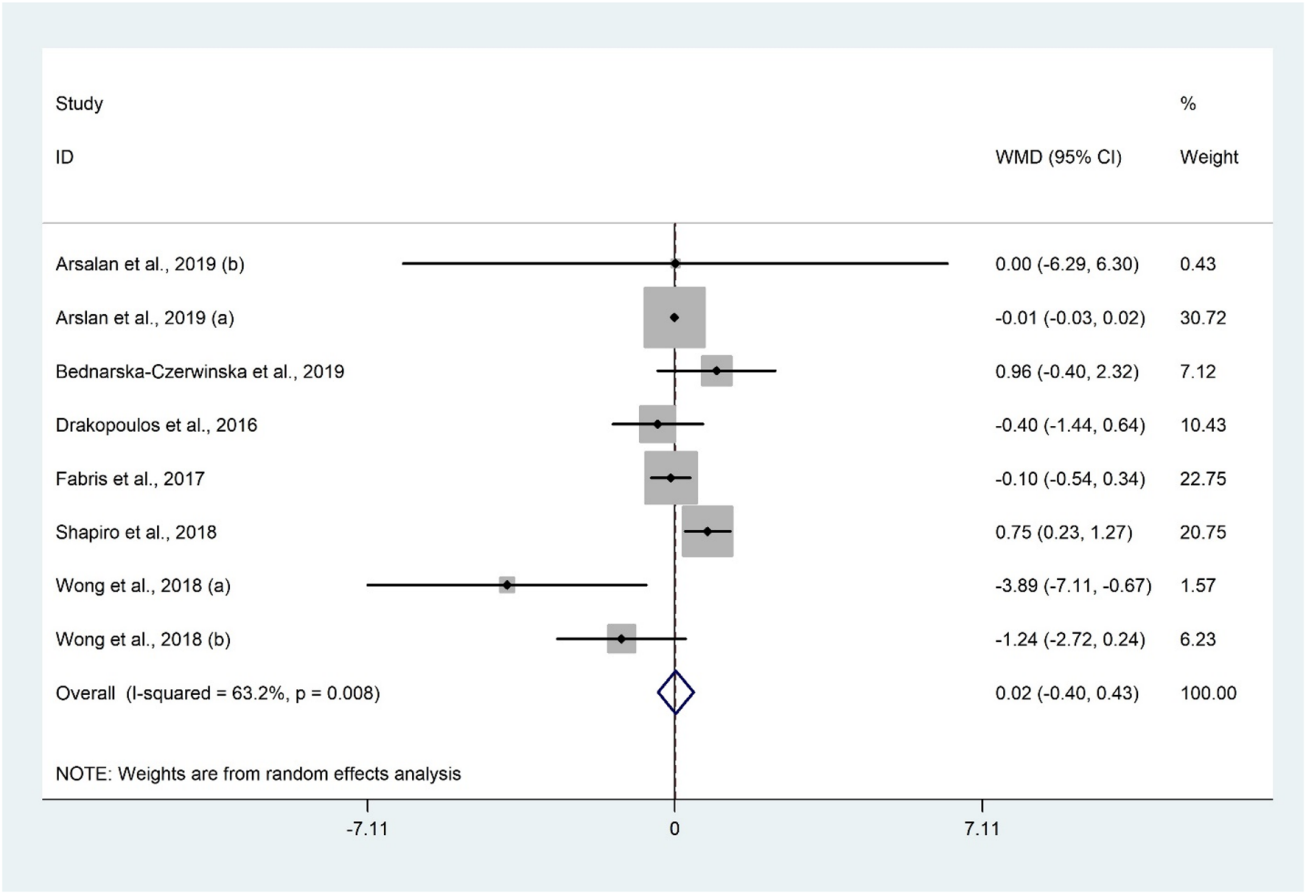
Forrest plot of the comparison of the AMH between women with vitamin D insufficiency/deficiency and sufficient ones.

Serum levels of AMH were compared between 1561 women with vitamin D insufficiency/deficiency and 924 women with sufficient vitamin D status using 8 studies^7,15,28,40,49,54^ yielded non-significant difference (WMD 0.02 ng/mL; 95% CI − 0.40 to 0.43; P = 0.929) with evidence of heterogeneity (I^2^ = 63.2%, P = 0.008) (Fig. 3). Furthermore, subgroup analysis did not change the results (Table 2). Meta-analysis findings were not sensitive to individual studies.

##### Serum 25(OH)D levels and FSH

**Figure 4:**
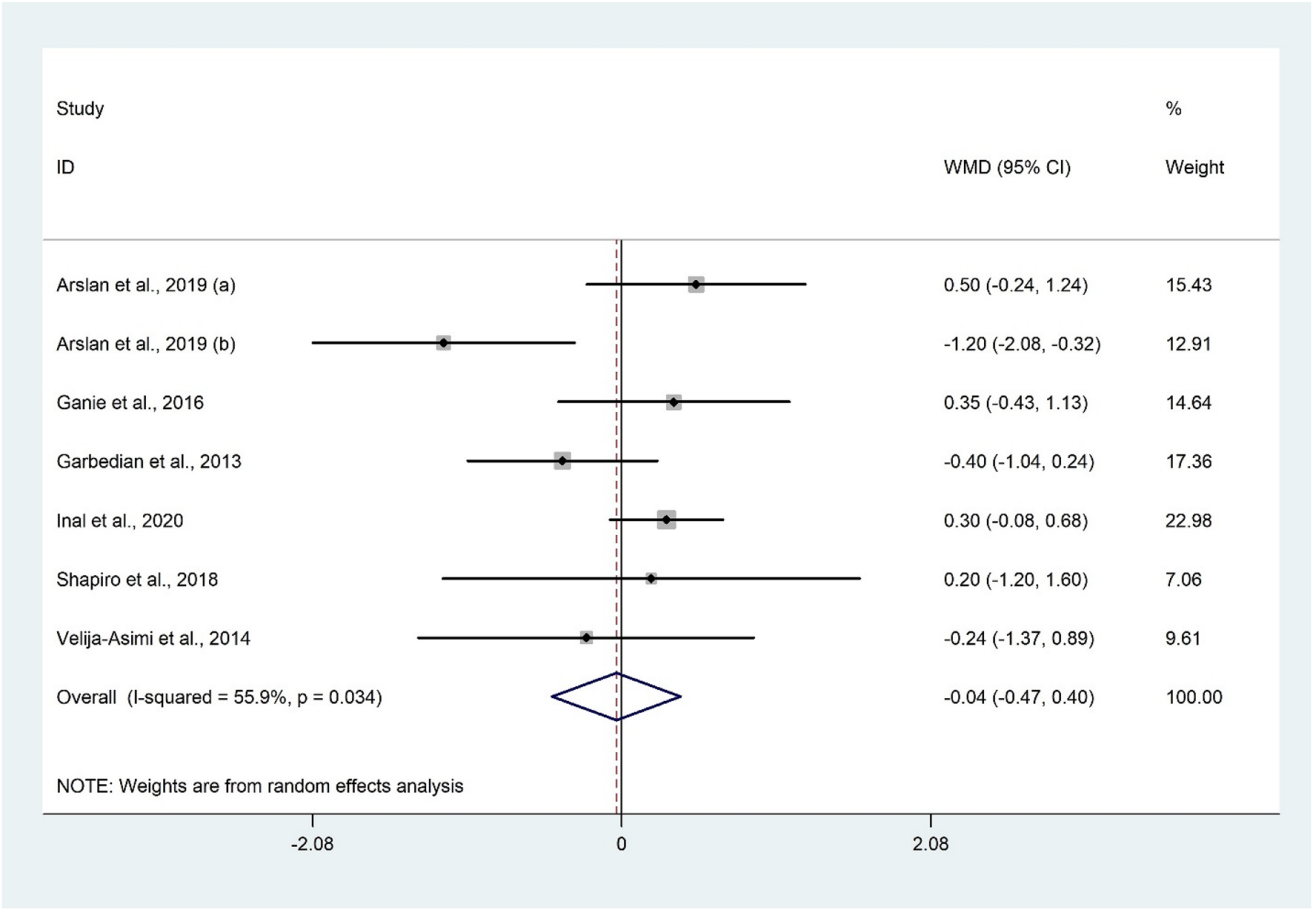
Forrest plot of the comparison of the FSH between women with vitamin D insufficiency/deficiency and sufficient ones.

The analysis of seven datasets^15,28,29,48,52,55^ including 1164 participants showed that serum FSH was not significantly associated with vitamin D status (WMD − 0.04 IU/l; 95% CI − 0.47 to 0.40; P = 0.870) with significant heterogeneity (I^2^ = 55.9%, P = 0.034) (Fig. 4). In addition, subgroup analysis did not change the findings (Table 2). The overall meta-analysis result for FSH was not sensitive to individual studies.

##### Serum 25(OH)D levels and LH

**Figure 5:**
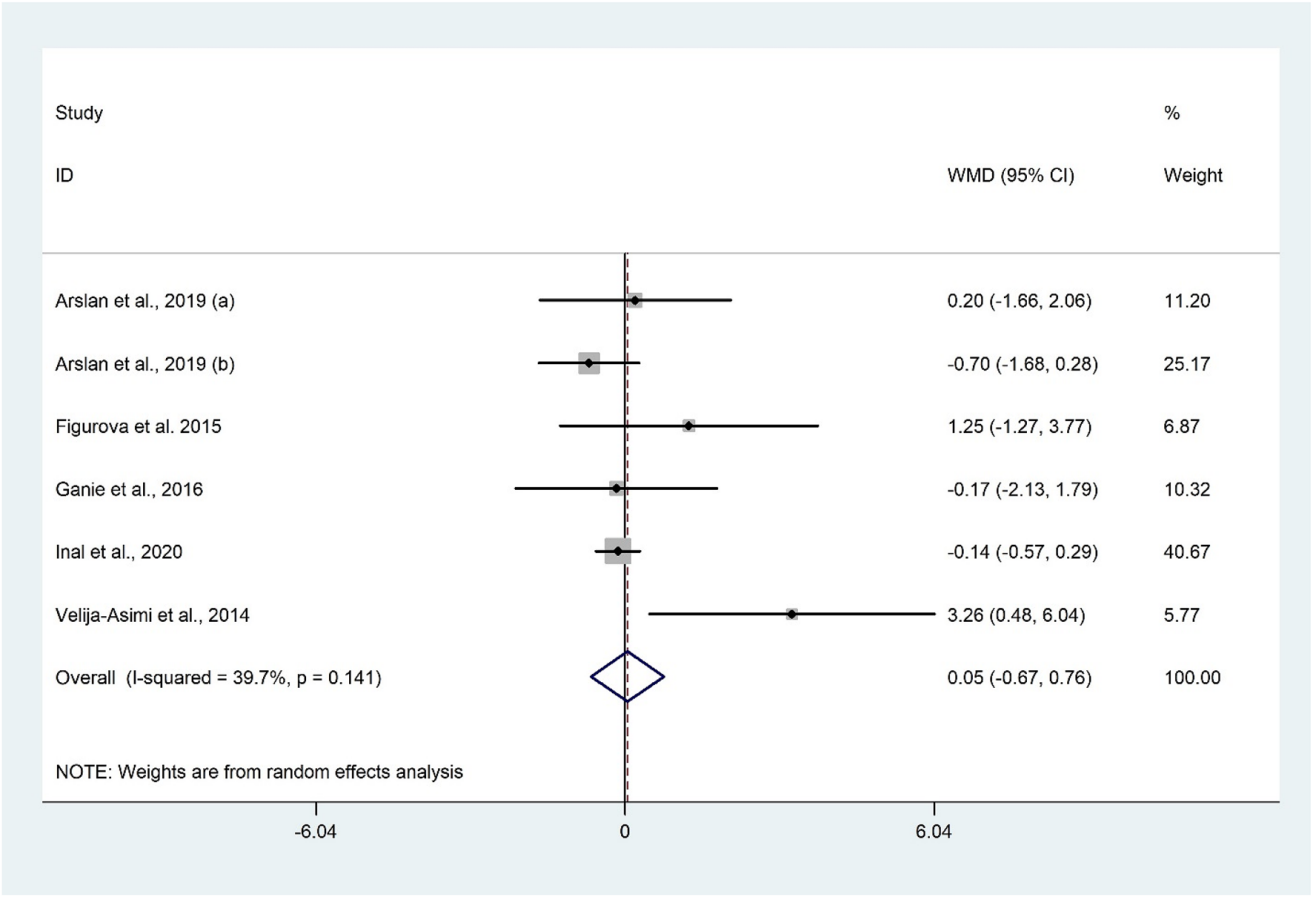
Forrest plot of the comparison of the LH between women with vitamin D insufficiency/deficiency and sufficient ones.

The difference of LH according to the vitamin D status was examined in six studies^28,29,48,50,52^ which was not significant (WMD 0.05 IU/l; 95% CI − 0.67 to 0.76; P = 0.900) (Fig. 5). Substantial heterogeneity was not observed (I^2^ = 39.7%, P = 0.141) among the included studies. Subgroup analysis of geographical areas revealed that serum LH was significantly higher among the non-Asian population with vitamin D insufficiency/deficiency compared to the control group (WMD 2.16 IU/l; 95% CI 0.20 to 4.12; P = 0.031) with no evidence of heterogeneity (I^2^ = 9.3%, P = 0.294) (Table 2). Excluding individual studies did not change the overall meta-analysis results.

#### Publication bias

No evidence of publication bias was observed for AFC (Begg’s test: P = 0.573, Egger’s test: P = 0.655), AMH (Begg’s test: P = 0.621, Egger’s test: P = 0.836), and FSH (Begg’s test: P = 0.293, Egger’s test: P = 0.401). There was evidence of publication bias for LH (Begg’s test: P = 0.039, Egger’s test: P = 0.251) and trim & fill analysis was applied. Two studies were filled and meta-analysis was done with new dataset but the results did not change (WMD − 0.23 ng/mL; 95% CI − 1.08 to 0.60; P = 0.577; I^2^ = 55.7%).

#### The correlation between ovarian reserve markers and serum 25(OH)D levels

##### The correlation between AFC and 25(OH)D levels

**Figure 6:**
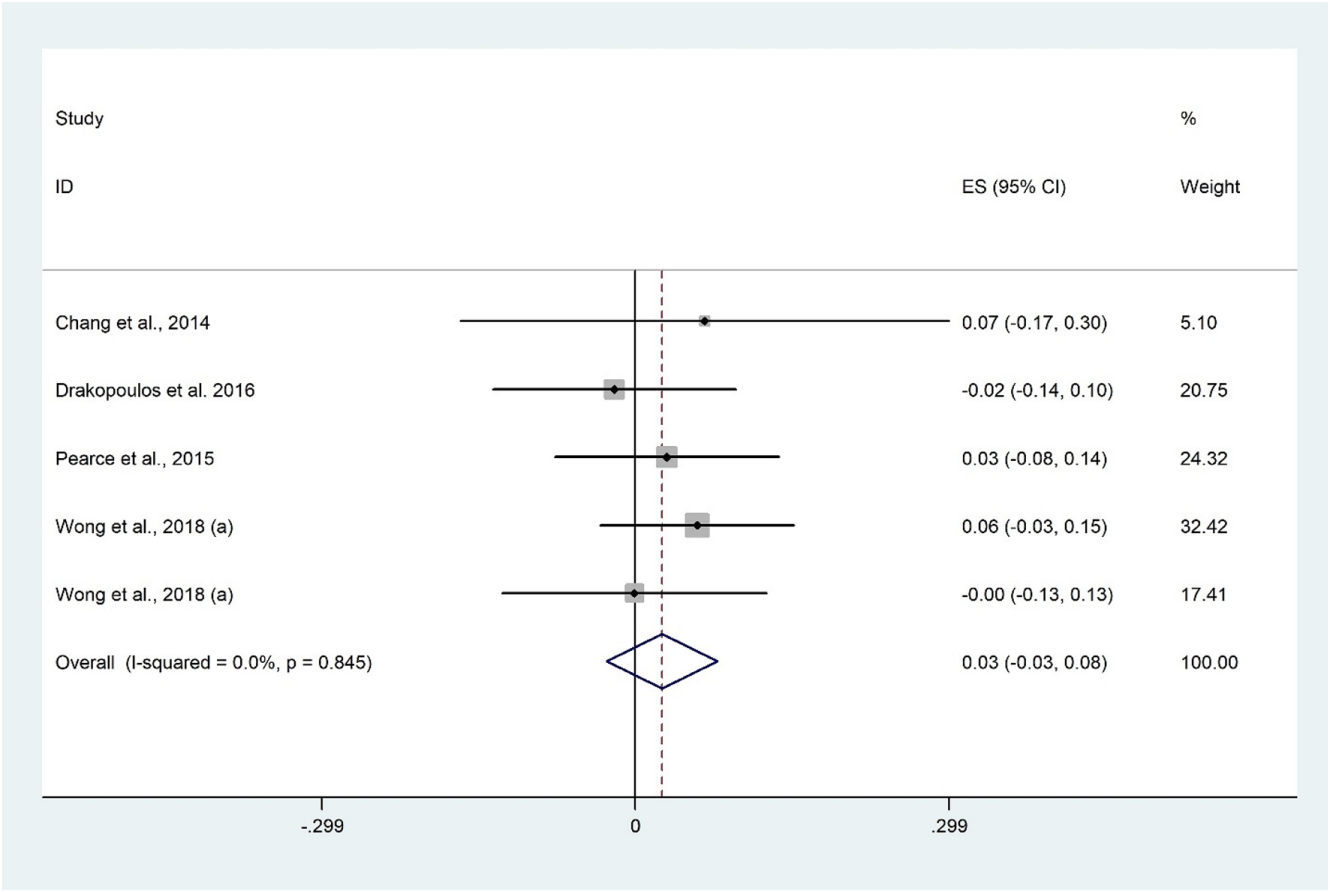
Forrest plot of the correlation between AFC and 25(OH)D level.

There was no significant correlation between AFC and 25(OH)D using 5 studies^7,14,53,54^ with 1391 participants (Fisher’s Z: 0.03; 95% CI − 0.03 to 0.08; P = 0.343) with no evidence of heterogeneity (I^2^ = 0.0%, P = 0.845) (Fig. 6). Findings were not sensitive to any individual studies.

##### The correlation between AMH and 25(OH)D levels

**Figure 7:**
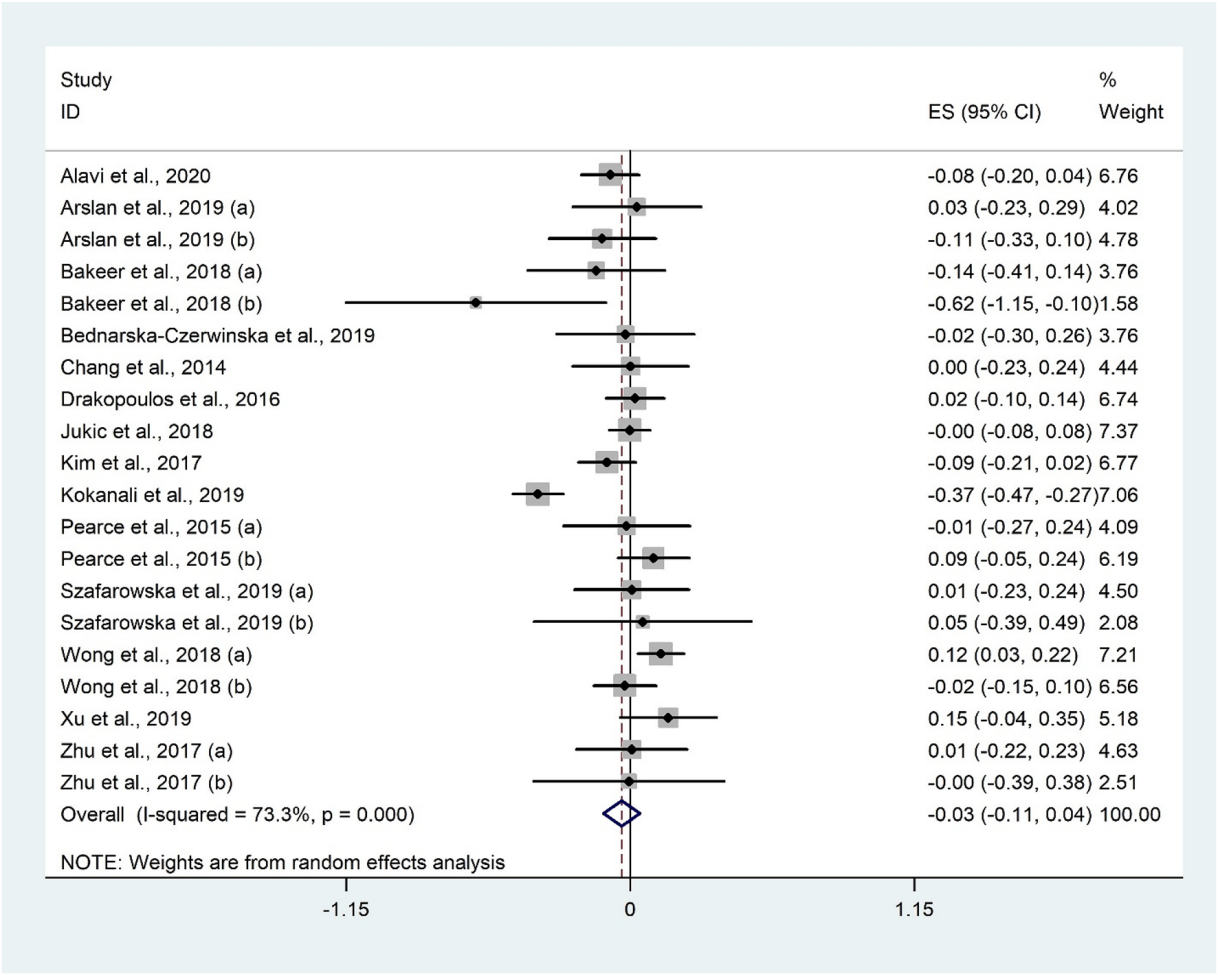
Forrest plot of the correlation between AMH and 25(OH)D level.

Twenty studies^7,14,28,32,34,37,40–42,45,46,51,53,54^ with 3406 subjects evaluated the correlation between AMH and 25(OH)D. There was no significant correlation between AMH and 25(OH)D (Fisher’s Z: − 0.03; 95% CI − 0.11 to 0.04; P = 0.355) with considerable heterogeneity (I^2^ = 73.3%, P < 0.001) (Fig. 7). Subgroup analysis was performed based on geographical areas, participants’ health status, and study design, however, the overall results did not change (Table 3). The overall meta-analysis result for AMH was not sensitive to individual studies.

**Table 3:** Subgroup analysis of the correlation between ovarian reserve markers and 25(OH)D level.

Sub-grouped by	No. of studies	Effect size^1^	95% CI	I^2^ (%)	P for heterogeneity	P for between subgroup heterogeneity
**AMH**						
Ovarian dysfunction						**0.048**
Without ovarian dysfunction	9	− 0.01	− 0.07, 0.04	14.8	0.310	
With ovarian dysfunction	10	− 0.05	− 0.18, 0.06	83.8	< 0.001	
With & without ovarian dysfunction	1	0.15	− 0.04, 0.34	–	–	
Geographical population						**0.278**
Asian	13	− 0.02	− 0.13, 0.07	81.1	< 0.001	
Non-Asian	7	− 0.01	− 0.08, 0.05	6.9	0.375	
Study design						** < 0.001**
Cross-sectional	13	− 0.02	− 0.08, 0.03	36.0	0.095	
Case–control	4	− 0.05	− 0.36, 0.25	89.4	< 0.001	
Cohort	3	0.01	− 0.05, 0.08	0.0	0.507	
**FSH**						
Ovarian dysfunction						** < 0.001**
Without ovarian dysfunction	3	0.03	− 0.12, 0.18	48.6	0.143	
With ovarian dysfunction	5	0.06	− 0.08, 0.21	69.0	0.012	
With & without ovarian dysfunction	5	− 0.25	− 0.55, 0.04	90.8	< 0.001	
Study design						**0.099**
Cross-sectional	7	− 0.13	− 0.40, 0.14	89.7	< 0.001	
Case–control	5	0.008	− 0.11, 0.13	62.9	0.029	
Cohort	1	0.01	− 0.07, 0.09	–	–	
**LH**						
Ovarian dysfunction						** < 0.001**
With ovarian dysfunction	6	− 0.004	− 0.16, 0.15	77.7	< 0.001	
With & without ovarian dysfunction	1	− 0.63	− 0.88, − 0.38	–	–	
Study design						**0.051**
Cross-sectional	3	− 0.13	− 0.61, 0.33	91.5	< 0.001	
Case–control	4	− 0.05	− 0.27, 0.16	86.2	< 0.001	
**LH/FSH ratio**						
Participants BMI						** < 0.001**
Overweight/obese	3	0.27	− 0.07, 0.61	83.3	0.003	
Normal	5	− 0.18	− 0.37, − 0.008	51.5	0.083	
Study design						**0.153**
Cross-sectional	4	− 0.007	− 0.48, 0.47	89.0	< 0.001	
Case–control	4	0.01	− 0.21, 0.24	80.8	0.001	

*AMH* anti-Mullerian hormone, *FSH* follicle stimulating hormone, *LH* luteinizing hormone, *BMI* body mass index.

^1^Calculated by Random-effects model as Fisher’s Z.

##### The correlation between FSH and 25(OH)D levels

**Figure 8:**
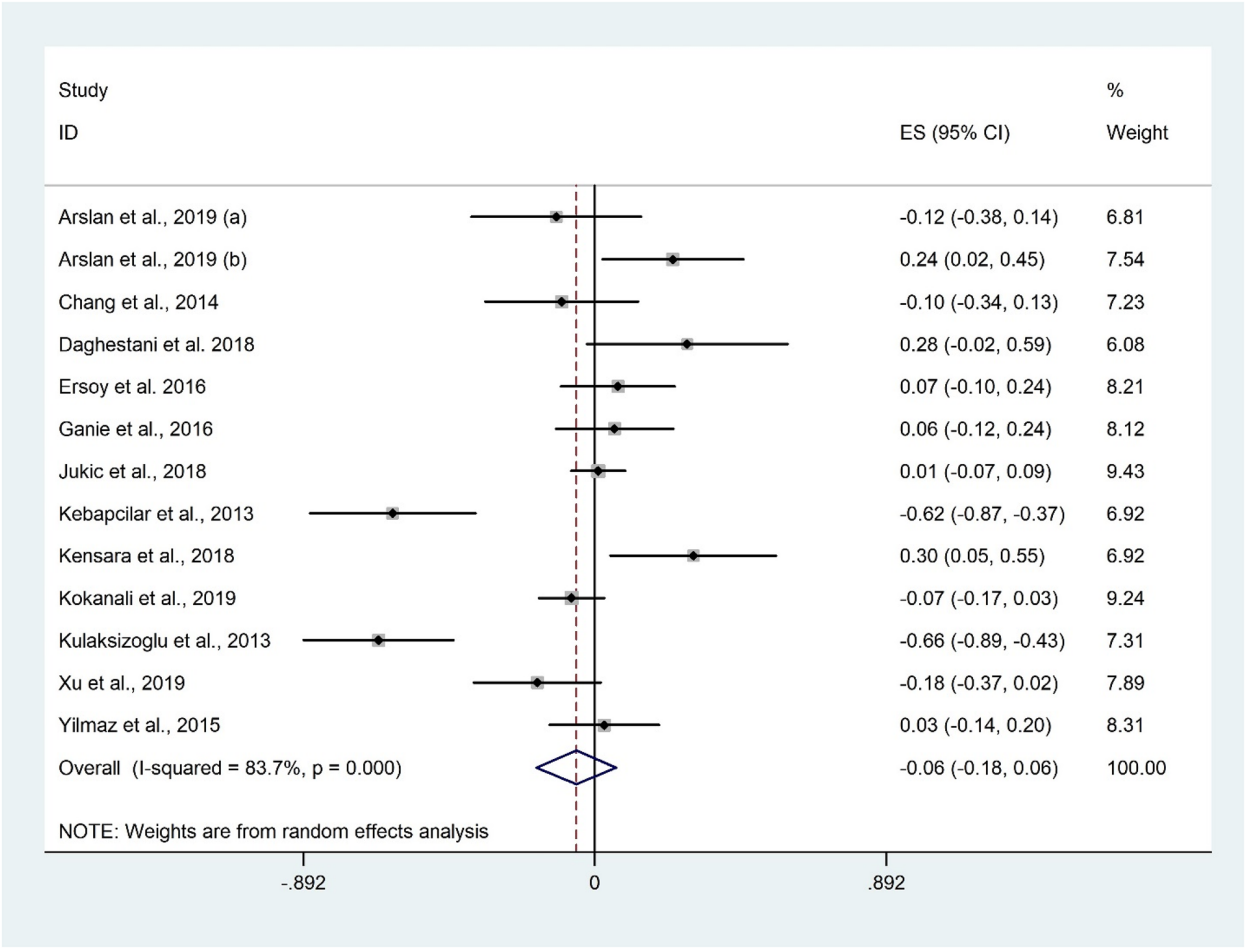
Forrest plot of the correlation between FSH and 25(OH)D level.

Correlation between FSH and 25(OH)D was observed in 13 studies^14,25–28,30,32,34,43–45,48^ with 1908 participants. Overall, there was no significant association between FSH and 25(OH)D (Fisher’s Z: − 0.06; 95% CI − 0.18 to 0.06; P = 0.357) (Fig. 8). There was evidence of substantial heterogeneity among the effect size of included studies (I^2^ = 83.7%, P < 0.001). Subgroup analysis based on participants’ health status and study design did not change the findings (Table 3). Excluding individual studies did not change the overall meta-analysis results.

##### The correlation between LH and 25(OH)D levels

**Figure 9:**
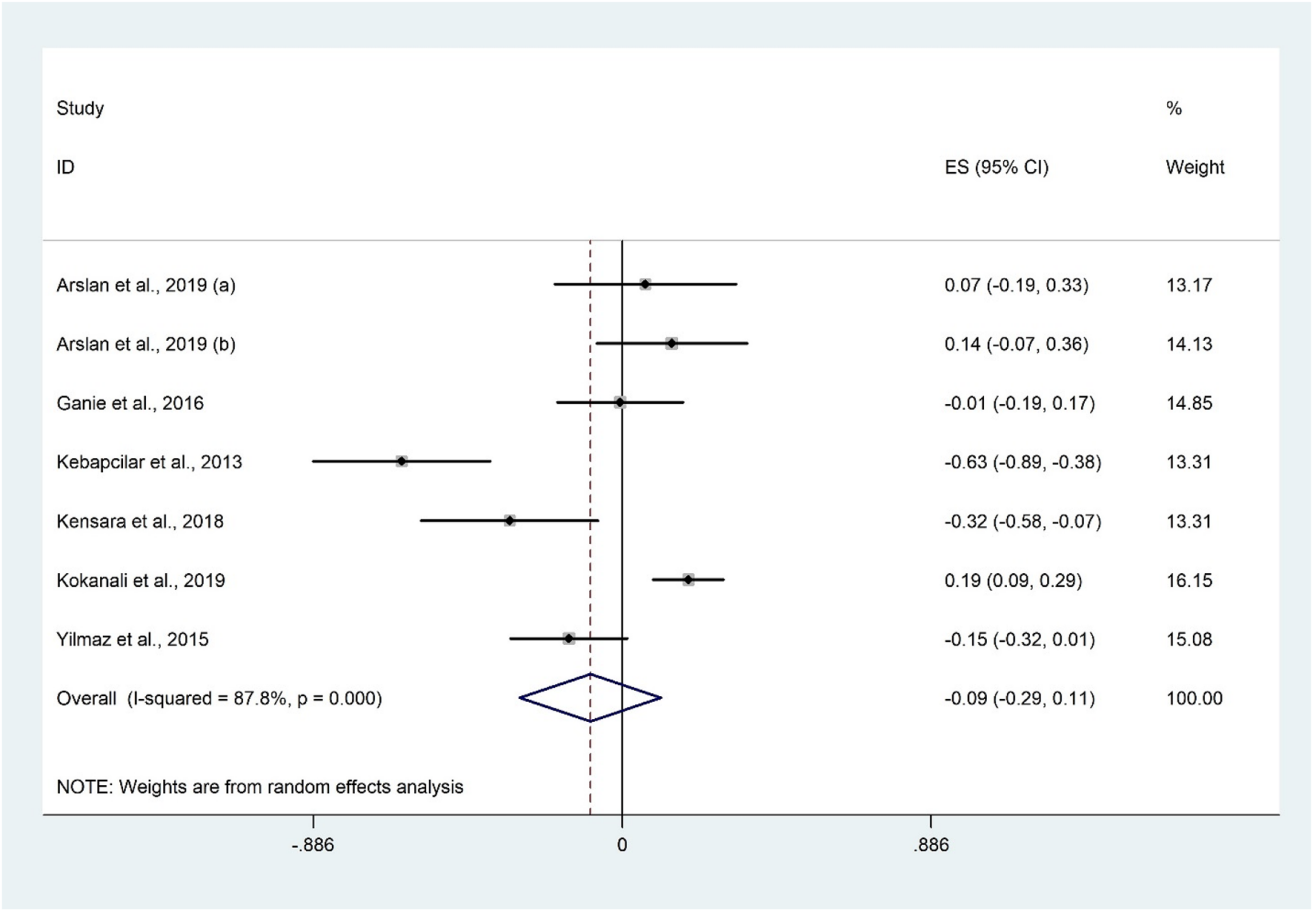
Forrest plot of the correlation between LH and 25(OH)D level.

The correlation between LH and vitamin D was not significant in the meta-analysis of seven studies^26,28,30,32,44,48^ with 919 participants (Fisher’s Z: − 0.09; 95% CI − 0.29 to 0.11; P = 0.372). Furthermore, evidence of significant heterogeneity was observed (I^2^ = 87.2%, P < 0.001) (Fig. 9). Subgroup analysis based on participants’ health status and study design did not change the overall findings (Table 3). Meta-analysis findings were not sensitive to individual studies.

##### The correlation between LH/FSH ratio and 25(OH)D levels

**Figure 10:**
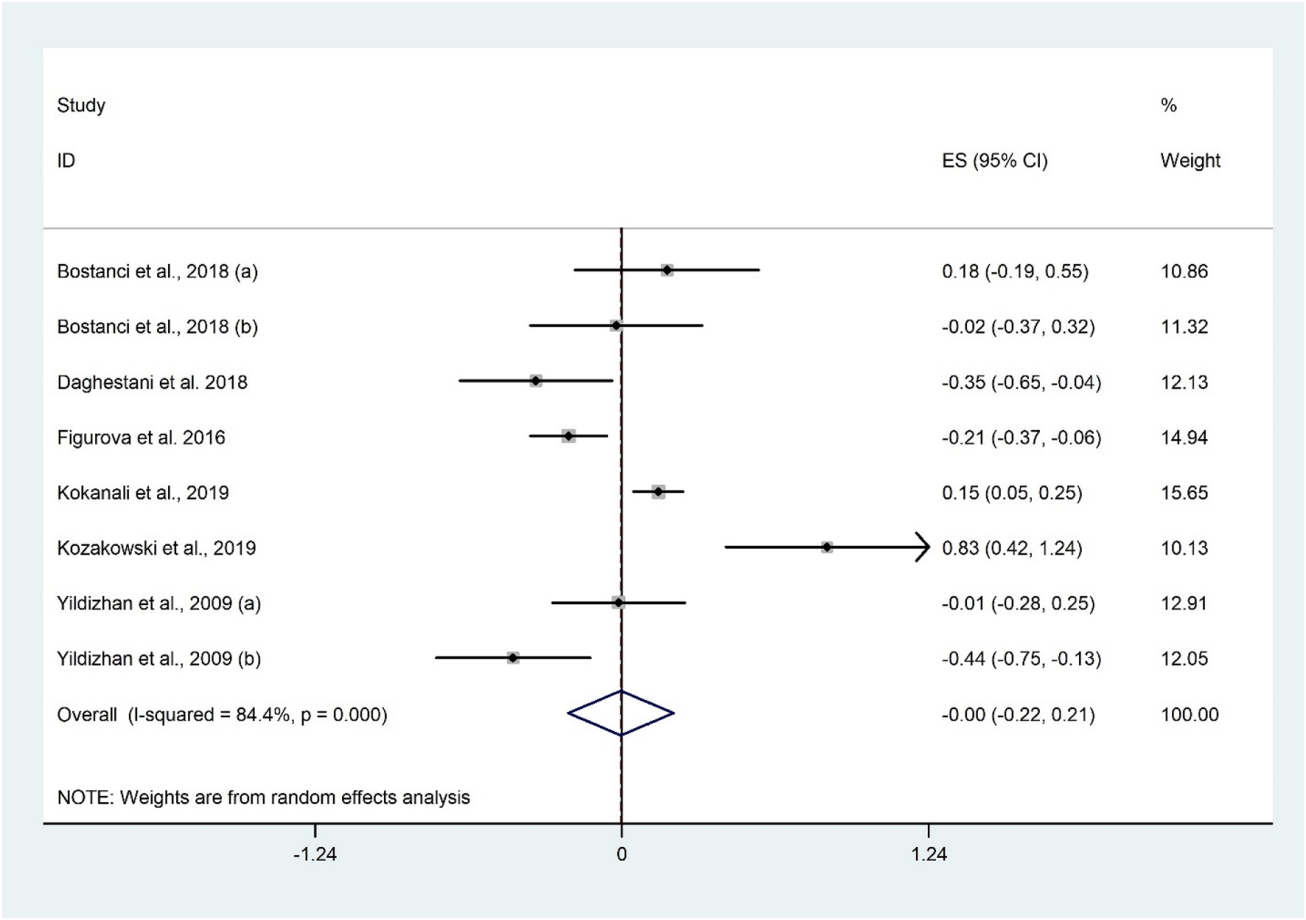
Forrest plot of the correlation between LH/FSH ratio and 25(OH)D level.

The correlation between LH/FSH ratio and 25(OH)D was examined in 8 studies^24,31,32,39,43,50^ with 786 participants. There was no significant association between LH/FSH ratio and 25(OH)D (Fisher’s Z: 0.004; 95% CI − 0.22 to 0.21; P = 0.971) with evidence of considerable heterogeneity (I^2^ = 84.4%, P < 0.001) (Fig. 10). Subgroup analysis revealed a negative correlation between LH/FSH ratio and 25(OH)D among women with normal BMI (Fisher’s Z: − 0.18; 95% CI − 0.37 to − 0.008; P = 0.041). The findings were not sensitive to any individual studies.

#### Publication bias

No evidence of publication bias was observed for AFC (Begg’s test: P = 0.624, Egger’s test: P = 0.911), AMH (Begg’s test: P = 0.381, Egger’s test: P = 0.990), FSH (Begg’s test: P = 0.951, Egger’s test: P = 0.651), LH (Begg’s test: P = 0.362, Egger’s test: P = 0.082), and LH/FSH ratio (Begg’s test: P = 0.216, Egger’s test: P = 0.751).

## Discussion

In order to identify new nutritional factors associated with women’s fertility, various attempts have been conducted. However, interpreting the literature to wrap up a conclusion is a difficult process for clinicians. Therefore, a comprehensive systematic review and meta-analysis of available literature can represent the most reliable evidence. Although previous systematic review and meta-analysis examined the relationship between concentrations of vitamin D and ovarian reserve^56^, that study focused only on AMH and included only 5 articles. Therefore, it was necessary to conduct a more comprehensive systematic review and meta-analysis on this relationship.

The present systematic review and meta-analysis of 36 observational studies examined the association between serum vitamin D levels and ovarian reserve markers including AMH, AFC, FSH, LH, and LH/FSH ratio in the adolescent and adult population of premenopausal women. Although, there was no significant association between serum vitamin D levels and any of the intended ovarian reserve markers, some of the subgroup analyses have found significant findings. AFC was significantly lower among Asians and LH was higher in the non-Asian population with vitamin D insufficiency/deficiency. Moreover, there was a negative correlation between vitamin D and LH/FSH ratio in women with normal BMI.

There are some points that should be taken into account when interpreting the results. First of all, there are substantial inter-assay differences in the performance of commercially available kits for serum vitamin D assay^57^. This notion may affect the results and play a considerable role as a heterogeneity factor. Additionally, seasonal variation in serum vitamin D should be considered when interpreting the results^58^. This notable fact has been excused in some papers^59,60^ and such inconsistency among the season of sample collection could also influence our final results. Lastly, there are several factors including race, skin color, use of skin protection (sunscreen), latitude, environmental pollution, aging, cultural and lifestyle issues that all can affect the synthesis and availability of vitamin D worthy to consider when interpreting the results^4,61,62^.

AMH is a glycoprotein hormone related to inhibin and activin and belongs to the family of transforming growth factor β (TGF-β), which has substantial functions in ovarian folliculogenesis^63^. AMH decreases follicle sensitivity to FSH^64^. Thus, there is absolute evidence that AMH is involved in the initiation of growth in follicles and FSH sensitivity^65^. The mechanism by which vitamin D may affect AMH and FSH is unclear. Similar to human studies, the findings of experimental studies regarding this association are also inconclusive^66–68^. Vitamin D may influence ovarian steroidogenesis, development of the follicles, and ovarian reserve^7^. AFC is a main prognosticator of the ovarian reserve and response to hormonal and follicle stimulation^69^. Furthermore, the related mechanism of vitamin D was regulated through VDR^70,71^, Thus, the ovarian reserve markers levels might be affected by the VDR polymorphism^72^. Interestingly, Szafarowska et al. reported that there is an association between polymorphisms of the VDR gene and AMH; however, they have not found any correlation between AMH levels and vitamin D concentrations in PCOS women^73^. On the other hand, some studies have represented that vitamin D deficiency and also single nucleotide polymorphism (SNP) of VDR did not affect dysmenorrhea, pelvic pain, or infertility^74^. Several studies have suggested that reduced vitamin D concentrations in PCOS and obese women may be associated with infertility^75,76^. A possible mechanism regarding the recent association might be decreased insulin sensitivity due to vitamin D deficiency^77^. Considering this hypothesis, insulin could elevate androgen biosynthesis and reduce sex hormone-binding globulin (SHBG) which resulted in hyperandrogenism. An excess amount of androgens is converted to estrogen. High estrogen concentration promotes the secretion of LH and represses FSH of the anterior pituitary^78^. Based on our findings, a negative correlation was observed regarding LH/FSH ratio with vitamin D suggesting that vitamin D status may contribute to hormonal dysregulation, even in women with normal BMI. According to the current meta-analysis, the issue that vitamin D levels are associated with ovarian reserve markers is still a controversial subject. Evidence is still unreliable as the randomized controlled trials are scarce, and the findings of available evidence are extremely heterogeneous.

On the other hand, the overall result of vitamin D and AFC showed a marginally significant association, whereas, the exclusion of Drakopoulos et al. and Fabris et al. studies from the analysis revealed a significant association between vitamin D and AFC. Based on the results of these studies, the change in results can be interpreted by Drakopoulos et al.'s study that was the only study to show that AFC was higher in women with vitamin D deficiency compared to those without vitamin D deficiency. In addition, Fabris et al.'s study demonstrated the least difference between the two groups with and without vitamin D deficiency in relation to AFC. As a result, the exclusion of these studies was able to make a significant result overall.

One of the substantial limitations in our study is the lack of evaluation of ethnicity in relation to vitamin D status, given that the vast majority of patients included in studies were Caucasian. Also, different methods of measuring vitamin D and the health status of the participants can be considered as other factors. Nevertheless, other parameters in the present meta-analysis were not sensitive to individual studies. The present meta-analysis had other limitations. There was significant heterogeneity in our study that may have affected the results and diminished the generalizability of the findings. The probable sources of heterogeneity might be differences in age, BMI, study design, vitamin D and ovarian reserves markers assay methods and kits, the season of sample collection, geographical variation, and the quality of the studies. Furthermore, the observational design of the included studies precludes us to examine the causality. Another limitation that may influence the findings is regarding vitamin D binding protein concentrations and VDR's polymorphisms that were not measured by the included studies. Moreover, seasonal variation of vitamin D was not taken into account in some of the included studies. In addition, the selected subgroup for the current study was not pre-specified that might be a source of bias and a limitation of the present systematic review and meta-analysis. Furthermore, the ovarian reserve mostly refers to the number and quality of dormant primordial follicles that cannot be explained completely by serum levels of AMH, AFC, LH, FSH, and LH/FSH ratio. On the other hand, these biochemical markers were selected as surrogate variables to illustrate the ovarian reserve. Another point to consider is the included studies did not use the same cut-off values for determining the patients’ vitamin D status. Moreover, there are potential confounders in the association between vitamin D and ovarian reserve including age, BMI, dietary intake, smoking, physical activity, and etc. Since most of the included studies did not comprehensively adjust for these confounders, this issue can influence our findings and should be considered as possible limitation.

## Conclusion

Based on what was discussed, although, there was no significant association between serum vitamin D levels and any of the intended ovarian reserve markers, some subgroup analyses have found significant findings. AFC was significantly lower among Asians and LH was higher in non-Asian population with vitamin D insufficiency/deficiency. Moreover, there was a negative correlation between vitamin D and LH/FSH ratio in women with normal BMI. In order to understand the underlying mechanisms of vitamin D in female reproduction, further attempts are needed.
